# Large-Scale Outbreak of *Mycoplasma pneumoniae* Infection, Marseille, France, 2023–2024

**DOI:** 10.3201/eid3007.240315

**Published:** 2024-07

**Authors:** Sophie Edouard, Housni Boughammoura, Philippe Colson, Bernard La Scola, Pierre-Edouard Fournier, Florence Fenollar

**Affiliations:** IHU–Méditerranée Infection, Marseille, France (S. Edouard, H. Boughammoura, P. Colson, B. La Scola, P.-E. Fournier, F. Fenollar);; Aix-Marseille Université, Marseille (S. Edouard, P. Colson, B. La Scola, P.-E. Fournier, F. Fenollar)

**Keywords:** *Mycoplasma pneumoniae*, outbreak, qPCR, pneumonia, co-infection, France, bacteria, respiratory infections

## Abstract

We report a large-scale outbreak of *Mycoplasma pneumoniae* respiratory infections encompassing 218 cases (0.8% of 26,449 patients tested) during 2023–2024 in Marseille, France. The bacterium is currently circulating and primarily affects children <15 years of age. High prevalence of co-infections warrants the use of a syndromic diagnostic strategy.

*Mycoplasma pneumoniae* is known to cause upper respiratory tract infections and pneumonia, especially in children 5–15 years of age (*1*). Although mostly sporadic, *M. pneumoniae* infections may occur as successive epidemics every few years (*1*). The precedent outbreak was observed during the 2019–2020 cold season, simultaneously in several countries, just before onset the COVID-19 pandemic (*2*). Then, the number of cases observed worldwide decreased markedly during this pandemic. However, although the resurgence of most respiratory pathogens was gradually observed from 2021, incidence of *M. pneumoniae* remained particularly low until June 2023, when a major resurgence of cases was reported worldwide (*2*–*3*).

We describe *M. pneumoniae* respiratory infections diagnosed in Marseille, France, university hospitals during January 1, 2014–February 15, 2024. We analyzed retrospectively all respiratory samples tested with 1 of the following specific quantitative PCRs (qPCRs) for *M. pneumoniae*: qPCR carried out by point-of-care laboratories using the Biofire FilmArray Respiratory Panel 2 Plus Assay (bioMérieux, https://www.biomerieux.com); qPCR performed routinely at the core laboratory using the FTD Respiratory Pathogens 21 Assay (Siemens Healthineers, https://www.siemens-healthineers.com); or an in-house specific qPCR (*4*). We used OpenEpi version 3.01 (https://www.openepi.com) for statistical analyses and considered differences significant at p<0.05.

Overall, 98,401 samples from 74,355 patients were tested for *M. pneumoniae* as part of the diagnosis of respiratory infections during 2014–2024. Median patient age was 30 years (range 0–108 years); 52% were male and 48% female. *M. pneumoniae* was detected in 449 patients (0.6%). Median age of positive patients was 10 years (range 0–101 years); 57% were male and 43% female.

We observed a few *M. pneumoniae* outbreaks in Marseille during 2014–2020, with a peak in early 2020 (Figure). Incidence then declined until a resurgence was observed beginning in 2023. Initially, 9 cases were observed in January 2023, followed by 6 cases during February–May. Then, a major increase in diagnoses was observed during June 1, 2023–February 15, 2024 (203 total with a peak of 48 cases in December 2023). From January 2023 through mid-February 2024, we diagnosed 218 *M. pneumoniae* infections (0.8% of 26,449 patients tested), compared with 231 cases (0.3% of 71,952 patients tested) during January 2014–December 2022 (p<0.0001). Median age was significantly lower for patients diagnosed since 2023 than for previous years (8 vs. 15 years; p<0.0001) (Table). Concurrent presence of >1 respiratory viruses was found for 114/316 (36%) *M*. *pneumoniae*–positive patients. The prevalence of co-infections was significantly higher in children <5 years of age than in other age groups (p<0.0001). The most common co-infections were with rhinovirus (n = 49), influenza A virus (n = 13), respiratory syncytial virus (n = 12), human coronavirus OC43 (n = 10), influenza B virus (n = 9) and metapneumovirus (n = 9) (Appendix Figure 1). Co-infections were significantly less frequent in patients diagnosed during 2014–2022 (45/164 [27%]) compared with those diagnosed since 2023 (69/152 [45.4%]; p = 0.0008).

**Figure:**
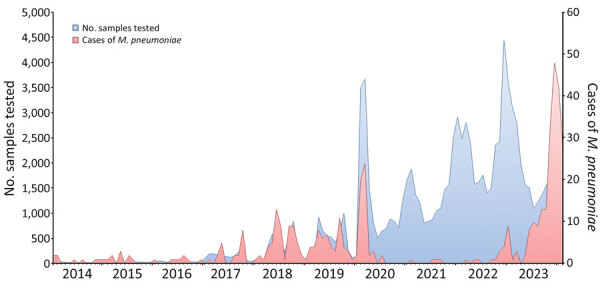
Monthly number of *Mycoplasma pneumoniae*–specific quantitative PCR tests performed and positive cases at a university hospital, Marseilles, France, January 1, 2014–February 15, 2024. Scales for the y-axes differ substantially to underscore patterns but do not permit direct comparisons.

**Table:** Demographic characteristics of 449 patients who had *Mycoplasma pneumoniae* infection diagnosed using quantitative PCR, Marseille, France, 2014–2022 versus 2023–2024*

Characteristic	Total		2014–2022		2023–2024		p value
No. patients	449		231		218		
Sex							
M	258 (57)		137 (59)		121 (56)		0.41
F	191 (43)		94 (41)		97 (44)		
Median age, y (range)	10 (0–101)		15 (0–93)		8 (0–101)		<0.001
Age group, y							
0–4	117 (26)		50 (22)		67 (31)		0.028
5–14	146 (32)		63 (27)		83 (38)		0.015
15–44	106 (24)		67 (29)		39 (18)		0.006
45–64	40 (9)		29 (13)		11 (5)		0.005
>65	40 (9)		22 (9)		18 (8)		0.638
Co-infections							
No. patients tested	316		164		152		
No. patients with co-infection	114 (36)		45 (27)		69 (45)		0.0008
With 1 pathogen	94 (82)		41 (91)		53 (77)		0.049
With >2 pathogens	20 (17)		4 (9)		16 (23)		
Median age, y (range)	4 (0–101)		7 (0–86)		3 (0–101)		0.012
Age group, y							
0–4	58 (51)		18 (40)		40 (58)		<0.001
5–14	27 (24)		7 (16)		20 (29)		0.005
15–44	13 (11)		10 (22)		3 (4)		0.089
45–64	8 (7)		6 (13)		2 (3)		0.286
>65	8 (7)		4 (9)		4 (6)		1

*Values are no. (%) except as indicated.

The increase of *M. pneumoniae* infection cases observed in our center are in line with observations from surveillance networks in France and throughout Europe (i.e., detection of the first epidemic sign in June 2023 until a peak reaching in December 2023) (*2*). High percentages of positivity (up to 50%) have been reported in China (*5*). In Marseille, we observed a lower percentage (1.8%), similar to the 0.89% observed in the United States since September 2023 (*6*). Most previous studies reported an increased incidence of *M. pneumoniae* infection particularly in school-age children and young adults (*3*,*7*). In Marseille, children <15 years were more affected during 2023–2024 than in previous seasons. However, we observed a switch regarding the population affected by the epidemic; adults became more affected beginning in January 2024 (Appendix Figure 2), possibly because of a massive transmission of the bacterium from infected children. We also observed a high rate of co-infections (≈50%), compared with 18% in the Netherlands (*3*). A high rate of co-infection with *M. pneumoniae* and other pathogens has also been previously reported in 65% of children and 34% of adults with acute respiratory infections in the United States (*8*). *M. pneumoniae* carriage ranging from 21% to 56% has also been reported in asymptomatic children (*9*). Interactions during co-detected microorganisms are complex, making it difficult to clearly define the contribution of each to respiratory infection. A high rate of asymptomatic carriers suggests a critical role for the nasopharyngeal microbiota in the clinical expression of respiratory infection.

There are several hypotheses for this re-emergence of *M. pneumoniae*, including the emergence of a new strain or a decline in individual and collective immunity. The current outbreak could be the usual periodic recurrence marked by an exacerbation resulting from a period of low exposure linked to restrictive measures during the COVID-19 pandemic. We did not investigate macrolide resistance, but reported resistance is low in Europe (*10*), and most studies described favorable outcomes after macrolide treatment (*7*). The number of *M. pneumoniae* infection cases is probably underestimated, particularly because patients with mild symptoms are not systematically tested. The high prevalence of co-infections with respiratory viruses justifies the use of a syndromic diagnostic strategy.

Appendix Additional information about large-scale outbreak of *Mycoplasma pneumoniae* infection, Marseille, France, 2023–2024.
