# Supplementary material for: Large-Scale Outbreak of Mycoplasma pneumoniae Infection, Marseille, France, 2023–2024
Source: Emerg Infect Dis. 2024 Jul;30(7):1481–4. doi: 10.3201/eid3007.240315 (PMC11210650; doi:10.3201/eid3007.240315)
Supplement: Appendix — Additional information about large-scale outbreak of Mycoplasma pneumoniae infection, Marseille, France, 2023–2024. [file 24-0315-Techapp-s1.pdf]

Article DOI: https://doi.org/10.3201/eid3007.240315

EID cannot ensure accessibility for supplementary materials supplied by authors. Readers who have difficulty accessing supplementary content should contact the authors for assistance.

Large-Scale Outbreak of *Mycoplasma pneumoniae* Infection, Marseille, France, 2023–2024

Appendix

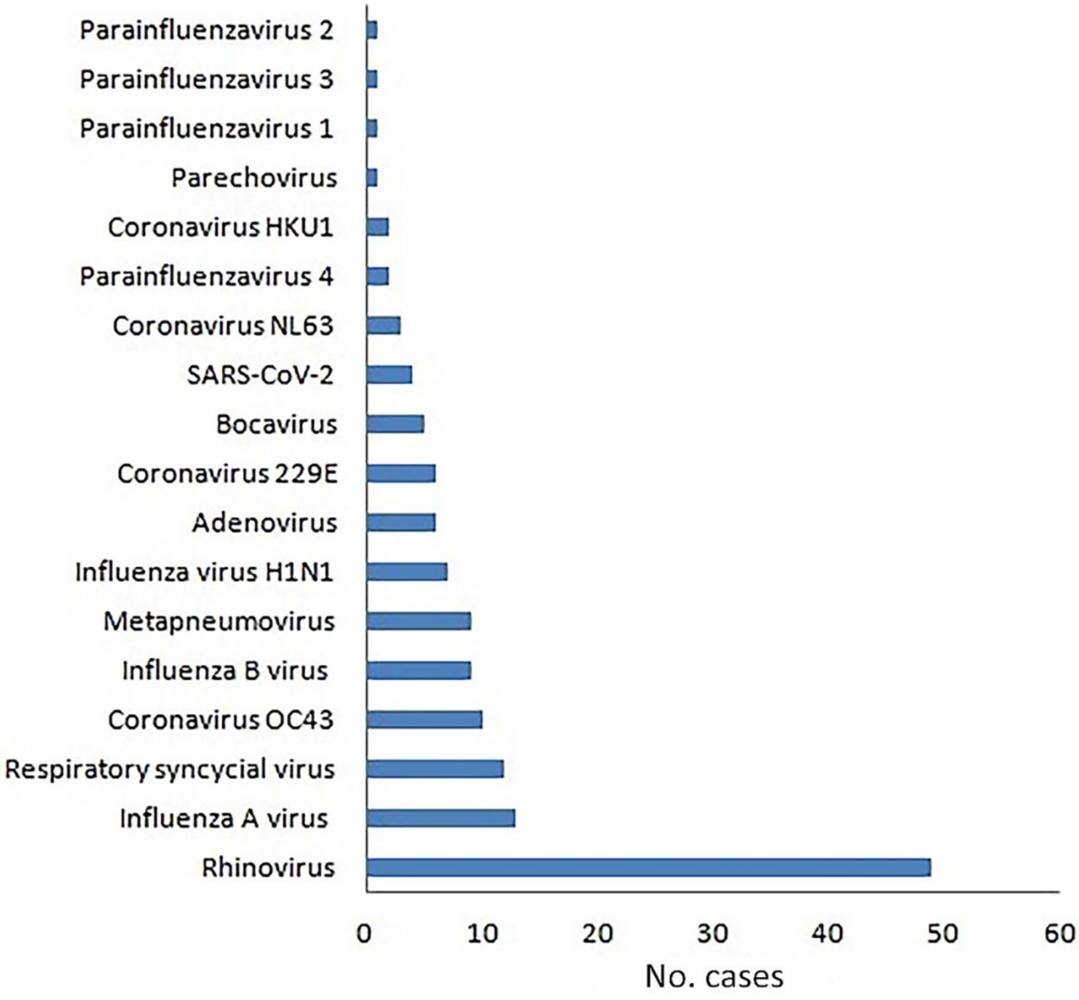

Appendix Figure 1. Coinfection of *M. pneumoniae* cases (n = 114), January 1, 2014–February 15, 2024.

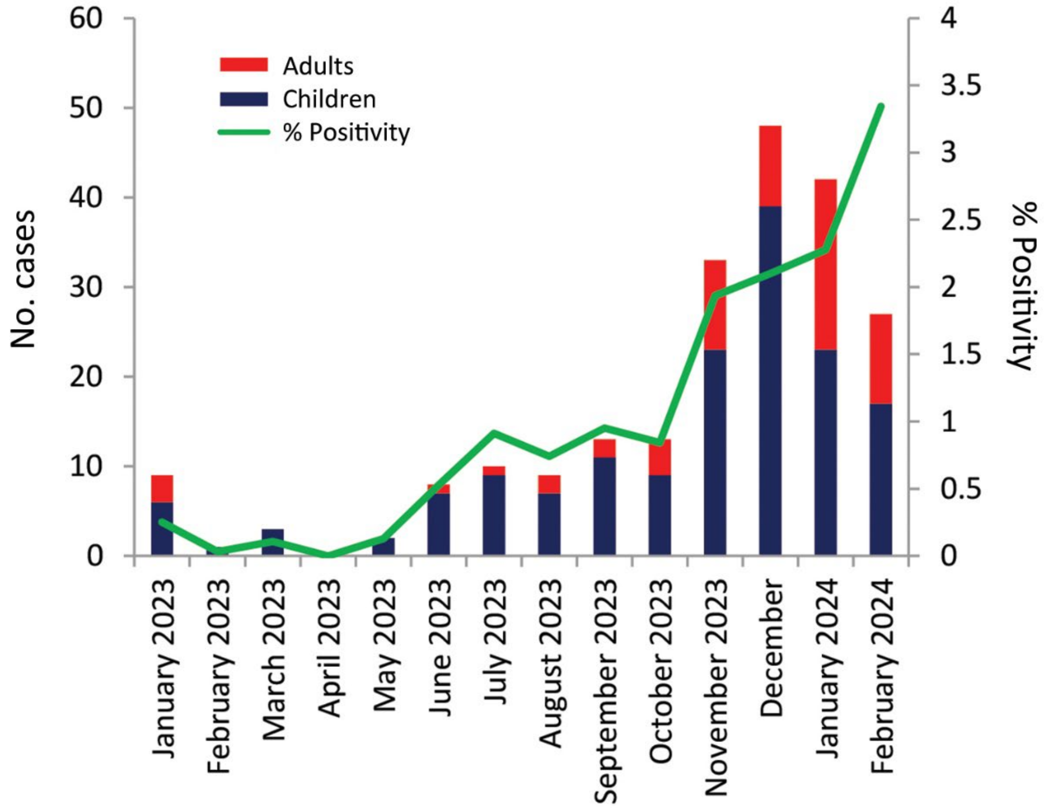

Appendix Figure 2. Number of children and adults infected with *M. pneumoniae* since January 2023.
